# Yangxin Tongluo Decoction Protects Against Sepsis‐Associated Cardiac Dysfunction Through Regulating Nrf2 Pathway

**DOI:** 10.1155/mi/2804249

**Published:** 2026-07-23

**Authors:** Hainan Yang, Beijing Wu, Die Huang, Hui Ye, Weifang Yuan, Lijiao You, Xueru Huang, Haikuo Wang, Yaoguo Han, Ming Lei

**Affiliations:** ^1^ Department of Critical Care Medicine, Seventh People’s Hospital of Shanghai University of Traditional Chinese Medicine, 358 Datong Road Pudong New District, Shanghai 200137, China, shutcm.edu.cn; ^2^ Department of Rehabilitation, Liuzao Community Health Service Center, Pudong New District, Shanghai 201322, China; ^3^ Department of General Practice, Beicai Community Health Service Center, Pudong New District, Shanghai 201204, China

**Keywords:** apoptosis, cardiac dysfunction, Nrf2 pathway, oxidative stress, sepsis, Yangxin Tongluo decoction

## Abstract

**Background:**

Sepsis‐induced cardiac dysfunction is a major cause of high mortality in critically ill patients. Yangxin Tongluo decoction (YXTLD), a traditional Chinese medicine (TCM) formula, is clinically used for such conditions, but its therapeutic effects and underlying mechanisms remain unclear. This study investigated the protective role of YXTLD against sepsis‐associated cardiac dysfunction and its relationship with the Nrf2 signaling pathway.

**Methods:**

An in vivo model of sepsis was established in male C57BL/6 mice via intraperitoneal lipopolysaccharide (LPS) injection. Cardiac function was assessed by echocardiography and serum biomarkers (creatine kinase [CK], lactate dehydrogenase [LDH]). In vitro, LPS‐stimulated H9c2 cardiomyocytes were treated with various doses of YXTLD. Cell viability, inflammation (interleukin [IL]‐1β, IL‐6, TNF‐α), oxidative stress (reactive oxygen species [ROS], malondialdehyde [MDA], catalase [CAT], glutathione [GSH]), and apoptosis were evaluated. The involvement of the Nrf2 pathway was examined using western blotting. In vivo dosage: YXTLD was administered intraperitoneally five times prior to LPS induction and continued after induction (total of seven doses over 48 h). In vitro concentrations: low (50 μg/mL), medium (100 μg/mL), and high (150 μg/mL) YXTLD.

**Results:**

YXTLD significantly improved cardiac function and alleviated myocardial injury in septic mice, as evidenced by increased ejection fraction (EF; 97.87% ± 1.20% vs. 79.05% ± 3.04%) and fractional shortening (FS; 74.99% ± 5.58% vs. 41.98% ± 2.72%), together with reduced serum CK (188.60 ± 81.16 vs. 566.75 ± 186.24 U/L) and LDH (179.70 ± 54.47 vs. 463.65 ± 197.58 U/L) levels compared with the LPS group (all *p*  < 0.05). In LPS‐stimulated H9c2 cells, YXTLD dose‐dependently enhanced cell viability, suppressed the expression of pro‐inflammatory cytokines (IL‐6, IL‐1β, and TNF‐α), reduced ROS production and lipid peroxidation, restored antioxidant capacity, and inhibited apoptosis. At 50 μg/mL, YXTLD markedly decreased IL‐6, IL‐1β, and TNF‐α levels by ~78%, 63%, and 92%, respectively (all *p* < 0.05). ROS and apoptotic cell proportions were progressively reduced with increasing YXTLD concentrations, reaching reductions of ~82% and 95%, respectively, at 150 μg/mL (*p* < 0.001). Mechanistically, YXTLD activated the Nrf2 signaling pathway by promoting nuclear Nrf2 translocation and upregulating its downstream antioxidant proteins, including heme oxygenase‐1 (HO‐1), NAD(P)H quinone dehydrogenase 1 (NQO1), and glutamate‐cysteine ligase modifier (GCLM). YXTLD also restored CAT and GSH levels while reducing MDA accumulation, indicating attenuation of oxidative stress.

**Conclusion:**

YXTLD protects against sepsis‐induced cardiac dysfunction by attenuating inflammation, oxidative stress, and apoptosis through activation of the Nrf2 signaling pathway. These findings provide a pharmacological basis for YXTLD as a potential therapeutic strategy for sepsis‐associated cardiac dysfunction.

## 1. Introduction

Sepsis, defined as systemic inflammatory response syndrome (SIRS), is an inappropriate immune reaction to infection that can trigger excessive inflammation [1, 2]. It is among the leading global causes of morbidity and mortality, with a high incidence and significant economic burden [3–8]. The heart is one of the major organ systems commonly affected by sepsis [9, 10]. Sepsis‐induced cardiac dysfunction, often presenting with reduced ejection fraction (EF), has been recognized and studied in both clinical and basic research for more than five decades, with reports dating back to 1951 [11]. Importantly, cardiovascular dysfunction in sepsis is associated with a markedly higher mortality rate—ranging from 70% to 90%—compared with about 20% in patients without cardiovascular impairment [12].

As research has advanced, multiple biomarkers have been identified in relation to sepsis‐associated myocardial dysfunction. For instance, well‐established indicators of myocardial ischemia include cardiac troponin I and T, as well as B‐type natriuretic peptide. Previous studies have demonstrated that elevated cardiac troponin levels correlate with the duration of hypotension and the intensity of vasopressor therapy [13, 14] and are also linked to poor short‐term outcomes [15, 16].

Nrf2 plays a central role in protecting cells from stress. Its activation helps prevent apoptosis and also regulates inflammatory responses by suppressing pro‐inflammatory cytokines [17, 18]. A small number of key inflammatory mediators act as central drivers of pathogenesis. Cytokines are major contributors to the myocardial inflammatory response, among which interleukins (ILs) are the most commonly involved inflammatory mediators [19]. For example, IL‐6 aggravates meningococcal septicemia‐induced myocardial depression by targeting p38 signaling in the mitogen‐activated protein kinase (MAPK) pathway [20, 21].

Accumulating evidence suggests that inhibition of inflammation‐related pathways, including TNF, IL‐1, oxidative stress, and apoptosis, can attenuate sepsis‐induced cardiomyocyte pyroptosis. However, most targeted therapeutic agents remain in the experimental stage [22]. For instance, STING deficiency alleviates lipopolysaccharide (LPS)‐induced septic cardiomyopathy by suppressing the STING–IRF3–NLRP3 axis and reducing inflammation, apoptosis, and pyroptosis [9]. Another study demonstrated that the receptor activator of nuclear factor kappa‐B ligand (RANKL) promotes inflammatory cytokine expression through the RANK–TRAF2/TRAF6–PLC–PKC–NF‐κB pathway, whereas blockade of RANKL signaling attenuates this inflammatory response during cardiac remodeling [23]. In addition, isopropyl 3‐(3,4‐dihydroxyphenyl)‐2‐hydroxypropanoate (IDHP) protects against LPS‐induced myocardial injury by reducing inflammation, oxidative stress, and apoptosis via activation of the GAS6/Axl–AMPK signaling pathway [24]. Similarly, amlodipine improves LPS‐induced cardiac dysfunction in septic rats by decreasing TNF‐α and iNOS levels through the activation of the PI3K/Akt pathway [25]. In recent years, increasing attention has been given to traditional Chinese medicine (TCM) for its significant therapeutic potential [26–28]. For instance, Huang et al. [29] reported that isoquercitrin, a bioactive component of *Gossypium herbaceum* L. and *Apocynum lancifolium* Rus., alleviated sepsis‐induced cardiac dysfunction by suppressing inflammation and promoting fatty acid oxidation via activation of AMPKα. Similarly, naringin was shown to reduce sepsis‐induced inflammation and myocardial injury through the regulation of the PI3K/AKT/NF‐κB pathway [30].

Although TCM is widely applied in the clinical management of sepsis‐induced cardiac dysfunction, there is still no clear evidence regarding the therapeutic effects of Yangxin Tongluo decoction (YXTLD), an empirical TCM formula composed of nine Chinese herbs, including Danggui (15 g), Danshen (15 g), Honghua (15 g), Chuanxiong (10 g), Huangqi (15 g), Gegen (15 g), Shengdi (15 g), Gualou (15 g), and Xiebai (15 g). Therefore, this study aimed to investigate whether YXTLD could alleviate cardiac insufficiency by modulating oxidative stress and inflammatory resistance through the Nrf2/heme oxygenase‐1 (HO‐1) signaling axis.

The novelty of the present study is that (1) this is the first investigation of YXTLD in sepsis‐associated cardiac dysfunction; (2) we provide the first mechanistic evidence linking YXTLD to activation of the Nrf2 signaling pathway; and (3) the study offers a pharmacological basis for future clinical translation.

## 2. Materials and Methods

### 2.1. Ethics Statement

All experimental animals (male C57BL/6 mice, 8–10 weeks old) were obtained from Shanghai SLAC (Shanghai, China). All procedures were conducted in accordance with institutional guidelines and approved by the Seventh People’s Hospital of the Shanghai University of Traditional Chinese Medicine. Before the experiments, the mice were housed at 25°C under a 12:12 h light–dark cycle for 1 week.

### 2.2. Preparation of YXTLD Extract

Herbal ingredients—Danggui (15 g), Danshen (15 g), Honghua (15 g), Chuanxiong (10 g), Huangqi (15 g), Gegen (15 g), Shengdi (15 g), Gualou (15 g), and Xiebai (15 g)—were weighed according to specified amounts. After soaking for 30 min, the herbs were extracted twice with boiling distilled water (30 and 20 min). The combined extracts were concentrated, filtered, and vacuum‐dried to obtain freeze‐dried YXTLD powder. Quality control: each batch of YXTLD powder was prepared under GMP‐like conditions. Batch‐to‐batch variability was maintained at <5% for all major marker compounds, consistent with previously reported quality control standards for TCM formulations [31, 32]. Extraction yield: The freeze‐dried powder yield was ~18.5% (w/w) of the raw herb weight.

### 2.3. LPS‐Induced Sepsis and YXTLD Treatment

Sepsis was induced in mice by an intraperitoneal injection of LPS (10 mg/kg; Sigma, MO, USA). YXTLD was administered via intragastric gavage five times prior to model induction. After sepsis induction, YXTLD administration via gavage was continued. Serum samples were collected 48 h after LPS induction.

### 2.4. Cardiac Function Evaluation

This study assessed cardiac function in mice by measuring cardiac biomarkers, including lactate dehydrogenase (LDH) and creatine kinase (CK), using an automatic biochemical analyzer (LABOSPECT 008 AS, Japan) according to the manufacturer’s instructions. In addition, transthoracic echocardiography was performed to evaluate the left ventricular (LV) function with an ultrasound system (VisualSonics, Toronto, Canada). Mice were anesthetized with 1.5%–2% isoflurane, and body temperature was maintained at ~37°C. LVLVEF and LV fractional shortening (LVFS) were then measured after LPS injection.

### 2.5. Cell Viability Assay

The Cell Counting Kit‐8 (CCK‐8, Dojindo, Kumamoto, Japan) assay was used to evaluate the dose‐dependent effects of YXTLD on H9c2 cell viability according to the manufacturer’s instructions. Briefly, H9c2 cells were seeded in 96‐well plates and treated with YXTLD at concentrations of 10, 20, 40, 80, 160, 320, 640, 1.28, and 2.56 mg/mL to determine the safe dose. Each condition was tested in five replicates.

### 2.6. Cell Culture and Treatment

H9c2 cells, obtained from the Cell Bank of the Chinese Academy of Sciences (Shanghai, China), were cultured in DMEM supplemented with 10% FBS and used for subsequent in vitro experiments. To mimic cell injury associated with sepsis‐induced cardiac dysfunction, H9c2 cells were exposed to LPS (1 μg/mL for 6 h) [33] and then randomly assigned to the following groups: (1) control group: cells treated with normal DMEM; (2) LPS group: cells treated with 1 μg/mL LPS for 6 h; (3) low‐dose YXTLD group (50 μg/mL): cells pretreated with YXTLD for 4 h, followed by 1 μg/mL LPS for 6 h; (4) medium‐dose YXTLD group (100 μg/mL): cells pretreated with YXTLD for 4 h, followed by 1 μg/mL LPS for 6 h; (5) high‐dose YXTLD group (150 μg/mL): cells pretreated with YXTLD for 4 h, followed by 1 μg/mL LPS for 6 h.

### 2.7. Evaluation of Inflammatory Response

This study examined inflammatory responses in vitro by measuring the expression of pro‐inflammatory cytokines. Total RNA was extracted from H9c2 cells using the TRIzol reagent (Invitrogen, Carlsbad, CA, USA) and reverse‐transcribed into cDNA with the PrimeScript RT reagent kit (TaKaRa, Otsu, Shiga, Japan) following the manufacturer’s instructions. RT‐qPCR was then performed to assess the expression levels of IL‐1β, IL‐6, and TNF‐α. The reactions were carried out using the SYBR Green I Master Mix kit (Invitrogen, Carlsbad, CA, USA) on a 7500 Real‐Time PCR System (Applied Biosystems, USA), with GAPDH serving as the internal control. Relative expression levels were calculated using the 2^−ΔΔCt^ method.

### 2.8. Detection of Reactive Oxygen Species (ROS)

Intracellular ROS in H9c2 cells was measured using DCFH‐DA (Nanjing Jiancheng, Nanjing, China) as previously described [34]. H9c2 cells were seeded at 10^4^ cells/mL and cultured at 37°C with 5% CO_2_ to achieve 60%–80% confluency. After washing twice with PBS, LPS‐induced injury was established with or without YXTLD. Cells were incubated with 10 μM DCFH‐DA in serum‐free DMEM at 37°C for 1 h. Fluorescence was observed with an Olympus DX51 microscope (Tokyo, Japan). Positive controls showed strong green fluorescence, and negatives were weak. Fluorescence intensity was quantified using ImageJ (NIH, Bethesda, MD, USA).

### 2.9. Malondialdehyde (MDA) and Glutathione (GSH) Measurement

Cardiomyocyte MDA levels were measured colorimetrically using assay kits as previously described, with absorbance read at 532 nm. Total and oxidized GSH (GSH and GSSG) concentrations were determined using a T‐GSH/GSSG Detection Kit following the manufacturer’s instructions.

### 2.10. Western Blotting

Total protein was extracted from treated cells using RIPA buffer (Beyotime, Shanghai, China). Proteins were quantified, separated by SDS‐PAGE, and transferred onto PVDF membranes. Membranes were blocked with 5% skimmed milk and incubated overnight at 4°C with primary antibodies: anti‐Nrf2 (1:1000, Abcam), anti‐HO‐1 (1:1000, Abcam), anti‐NQO1 (NAD(P)H quinone dehydrogenase 1) (1:1000, Abcam), anti‐GCLM (glutamate‐cysteine ligase modifier) (1:1000, Cell Signaling Technology), and anti‐GAPDH (1:1000, Abcam). After incubation with secondary antibodies, protein bands were visualized using an ECL chemiluminescent reagent (Beyotime).

### 2.11. Statistical Analysis

Statistical analyses were performed using SPSS 22.0 (SPSS Inc., Chicago, IL) and GraphPad Prism 7.0 (GraphPad Software, USA). Data are presented as the mean ± SD. Differences between groups were evaluated by Student’s *t*‐test or one‐way ANOVA with Tukey’s post hoc test.

## 3. Results

### 3.1. YXTLD Improves Cardiac Function in Septic Mice

**Figure 1:**
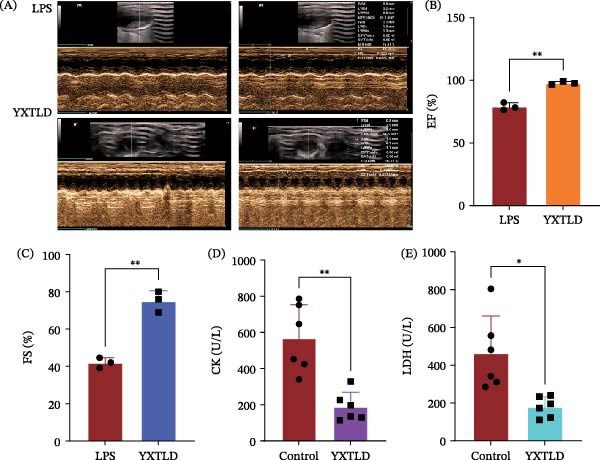
Echocardiographic function and myocardial injury markers in LPS‐induced sepsis. (A) Representative echocardiographic images. (B) Left ventricular ejection fraction (LVEF). (C) Left ventricular fractional shortening (LVFS). (D, E) Serum CK and LDH levels in LPS‐induced mice. Data are presented as mean ± SD (*n* = 3–6).  ^∗^ *p* < 0.05,  ^∗∗^ *p* < 0.01 vs. control group.

To evaluate the therapeutic effects of YXTLD in septic mice, echocardiographic parameters and serum myocardial injury markers, including CK and LDH, were assessed 48 h after LPS‐induced sepsis. Echocardiographic analysis demonstrated that YXTLD treatment significantly improved cardiac function, as evidenced by increases in EF (79.05% ± 3.04% vs. 97.87% ± 1.20%) and FS (41.98% ± 2.72% vs. 74.99% ± 5.58%) compared with the LPS group (Figure 1A–C, *p* < 0.01). Furthermore, YXTLD treatment markedly reduced serum CK levels (566.75 ± 186.24 vs. 188.60 ± 81.16 U/L) and LDH levels (463.65 ± 197.58 vs. 179.70 ± 54.47 U/L) relative to the LPS group, indicating attenuation of myocardial injury (both *p* < 0.05; Figure 1D, E). Collectively, these findings demonstrate that YXTLD effectively ameliorates sepsis‐induced cardiac dysfunction by improving cardiac performance and reducing myocardial injury.

### 3.2. YXTLD Enhances Cell Viability and Reduces Inflammation in LPS‐Treated Cardiomyocytes

**Figure 2:**
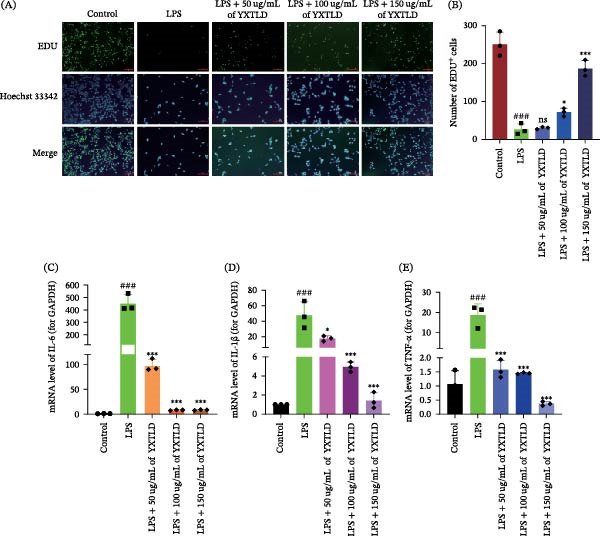
YXTLD enhances cell viability and attenuates inflammation in LPS‐treated cardiomyocytes. (A, B) YXTLD improved the viability of H9c2 cells. (C–E) YXTLD suppressed the LPS‐induced increases in IL‐1β, IL‐6, and TNF‐α mRNA expression in H9c2 cells. Data are presented as mean ± SD. ^###^ *p* < 0.001 vs. control group;  ^∗^ *p* < 0.05,  ^∗∗^ *p* < 0.01,  ^∗∗∗^ *p* < 0.001 vs. LPS group.

To evaluate the anti‐inflammatory effects of YXTLD in vitro, three concentrations of YXTLD, selected based on the results of the CCK‐8 assay (Figure S1), were tested in the mouse cardiomyocyte cell line H9c2. Following LPS stimulation, H9c2 cell proliferation was markedly inhibited, accompanied by significant increases in the expression of the pro‐inflammatory cytokines IL‐1β, IL‐6, and TNF‐α (all *p* < 0.001), confirming the successful establishment of an inflammatory cardiomyocyte injury model. YXTLD treatment significantly improved H9c2 cell viability in a dose‐dependent manner. While the low‐dose treatment (50 μg/mL) produced only a minimal effect, the medium‐dose (100 μg/mL) and high‐dose (150 μg/mL) treatments significantly restored cell viability compared with the LPS group (*p* < 0.05; Figure 2A, B). Furthermore, YXTLD markedly attenuated the inflammatory response by reducing the LPS‐induced upregulation of IL‐6, IL‐1β, and TNF‐α mRNA expression (Figure 2C–E). Specifically, treatment with 50 μg/mL YXTLD significantly decreased IL‐6 expression from 453.68 ± 68.82 in the LPS group to 97.62 ± 12.34, IL‐1β expression from 47.79 ± 17.43 to 17.72 ± 3.50, and TNF‐α expression from 18.62 ± 5.64 to 1.58 ± 0.32 (all *p* < 0.05), with medium‐ and high‐dose YXTLD producing comparable or greater anti‐inflammatory effects.

### 3.3. YXTLD Alleviates LPS‐Induced Oxidative Injury in H9c2 Cardiomyocytes

**Figure 3:**
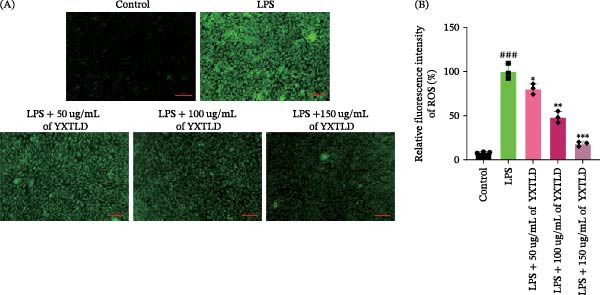
(A) ROS activation in H9c2 cardiomyocytes after LPS stimulation and its attenuation by YXTLD. Intracellular ROS was measured using the DCFH‐DA (2^′^,7^′^‐dichlorodihydrofluorescein diacetate) probe after LPS treatment. (B) Data are expressed as mean ± SD from at least three independent experiments. ^###^ *p* < 0.001 vs. control group;  ^∗^ *p* < 0.05,  ^∗∗^ *p* < 0.01,  ^∗∗∗^ *p* < 0.001 vs. LPS group.

To further investigate the anti‐inflammatory effects of YXTLD on H9c2 cells, intracellular ROS levels were evaluated using a fluorescent ROS probe. Following LPS stimulation, ROS production was markedly increased (*p* < 0.001), confirming the successful establishment of an oxidative injury model in cardiomyocytes. Notably, YXTLD significantly attenuated ROS generation in a dose‐dependent manner. Compared with the LPS group (mean ± SD = 100.00 ± 8.64), ROS levels were significantly reduced following treatment with YXTLD at 50 μg/mL (80.46 ± 5.91, *p* < 0.05), 100 μg/mL (48.45 ± 6.49, *p* < 0.01), and 150 μg/mL (18.09 ± 2.57, *p* < 0.001) (Figure 3).

### 3.4. YXTLD Attenuates Apoptosis in H9c2 Cardiomyocytes

**Figure 4:**
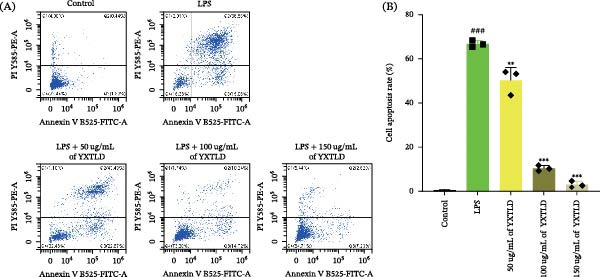
YXTLD attenuates apoptosis in H9c2 cardiomyocytes. (A) Flow cytometry analysis of H9c2 cardiomyocytes treated with three different concentrations of YXTLD. (B) YXTLD reverses the increased proportion of annexin V‐positive and PI‐positive H9c2 cells. Data are presented as mean ± SD. ^###^ *p* < 0.001 vs. control group;  ^∗∗^ *p* < 0.01,  ^∗∗∗^ *p* < 0.001 vs. LPS group.

LPS stimulation significantly increased the proportion of annexin V‐ and PI‐positive H9c2 cells, confirming the successful establishment of an injured cardiomyocyte model. YXTLD treatment markedly attenuated LPS‐induced apoptosis in a dose‐dependent manner, as evidenced by the progressive reduction in the percentage of annexin V‐ and PI‐positive cells (Figure 4). Compared with the LPS group (66.81 ± 1.43%), the apoptotic cell proportion was significantly decreased following treatment with 50 μg/mL YXTLD (50.24% ± 5.86%, *p* < 0.01), 100 μg/mL YXTLD (10.42% ± 1.29%, *p* < 0.001), and 150 μg/mL YXTLD (3.06% ± 1.48%, *p* < 0.001), demonstrating a clear dose‐dependent antiapoptotic effect.

### 3.5. YXTLD Administration Attenuates Oxidative Stress in H9c2 Cardiomyocytes by Activating the Nrf2 Signaling Pathway

**Figure 5:**
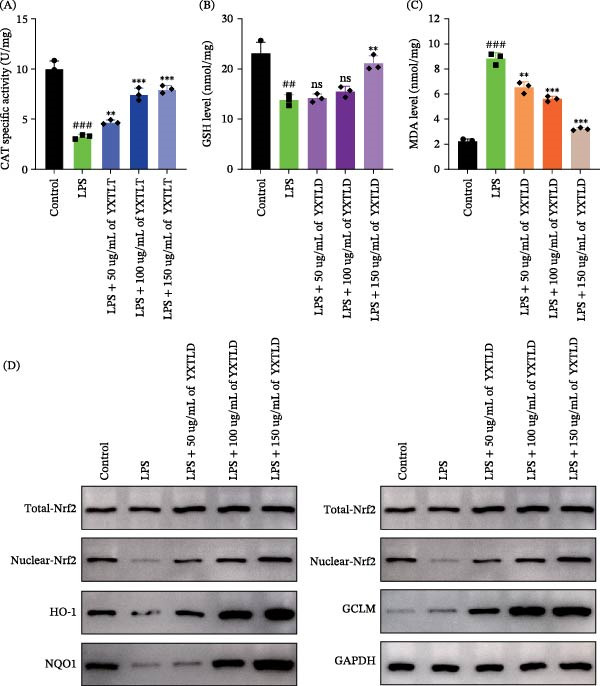
Regulatory effect of the Nrf2 signaling pathway on oxidative stress in H9c2 cardiomyocytes. (A) CAT content in H9c2 cardiomyocytes. (B) GSH content in H9c2 cardiomyocytes. (C) MDA content in H9c2 cardiomyocytes. (D) Western blot analysis of total Nrf2, nuclear Nrf2, HO‐1, NQO1, and GCLM in H9c2 cardiomyocytes. Data are presented as mean ± SD from at least three independent experiments. ^##^ *p* < 0.01, ^###^ *p* < 0.001 vs. control group;  ^∗∗^ *p* < 0.01,  ^∗∗∗^ *p* < 0.001 vs. LPS group.

LPS stimulation significantly decreased the levels of the antioxidant enzymes CAT (catalase) and GSH in H9c2 cardiomyocytes, indicating impaired antioxidant capacity. YXTLD treatment effectively reversed these changes. Specifically, the low‐dose YXTLD group (50 μg/mL) significantly increased CAT levels compared with the LPS group (*p* < 0.01), whereas the medium‐dose (100 μg/mL) and high‐dose (150 μg/mL) groups produced a more pronounced increase (both *p* < 0.001) (Figure 5A). CAT levels increased from 3.25 ± 0.21 in the LPS group to 4.70 ± 0.21 in the low‐dose YXTLD group. In contrast, low‐ and medium‐dose YXTLD exerted only modest effects on GSH levels, whereas the high dose (150 μg/mL) significantly restored GSH levels (*p* < 0.01), increasing them from 13.80 ± 1.05 in the LPS group to 21.13 ± 1.54 (Figure 5B). Consistent with these findings, LPS stimulation markedly increased MDA levels, indicating enhanced lipid peroxidation. YXTLD treatment significantly reduced MDA accumulation, with a significant decrease observed even at the low dose (50 μg/mL, *p* < 0.01) (Figure 5C). MDA levels decreased from 8.84 ± 0.47 in the LPS group to 6.54 ± 0.44 following treatment with 50 μg/mL YXTLD.

To further investigate whether Nrf2 is a molecular target of YXTLD, the protein expression of nuclear Nrf2 and its downstream antioxidant effectors, NQO1, HO‐1, and GCLM subunit, was examined by western blotting. LPS stimulation markedly reduced the expression of nuclear Nrf2 and its downstream proteins, whereas YXTLD treatment significantly restored their expression (Figure 5D). Collectively, these findings indicate that YXTLD alleviates LPS‐induced oxidative stress in H9c2 cardiomyocytes, at least in part, through the activation of the Nrf2 signaling pathway.

## 4. Discussion

In this study, we demonstrated that YXTLD exerts a protective effect against sepsis‐induced cardiac dysfunction in both in vivo and in vitro experiments. The main findings are as follows: (1) YXTLD improves cardiac function in septic mice; (2) YXTLD enhances cell viability and reduces inflammation in LPS‐treated cardiomyocytes; (3) YXTLD alleviates LPS‐induced oxidative injury in H9c2 cardiomyocytes; (4) YXTLD attenuates apoptosis in H9c2 cardiomyocytes; and (5) YXTLD mitigates oxidative stress in H9c2 cardiomyocytes via activation of the Nrf2 signaling pathway.

Sepsis is a dysregulated host response to infection that leads to the dysfunction of multiple organs, including the cardiovascular system [35]. Cardiac insufficiency is a major contributor to multiple organ failure and mortality in intensive care units [36–38]. Inflammatory cytokines, such as IL‐1β, IL‐6, and TNF‐α, are markedly elevated during sepsis and play a key role in driving organ dysfunction [39–41].

In China, the use of natural plants for medicinal purposes has a long‐standing tradition, and TCM is widely applied in the clinical treatment of various diseases [27, 28]. For example, Yunvjian decoction alleviates LPS‐induced acute lung injury by suppressing the NF‐κB/NLRP3 signaling pathway and subsequent pyroptosis in lung epithelial cells and macrophages [26]. Consistent evidence has shown that matrine markedly improves cardiac function and attenuates inflammation in sepsis models both in vivo and in vitro, with its cardioprotective effects mediated via the PTENP1/miR‐106b‐5p signaling axis [33]. Similarly, honokiol has been demonstrated to enhance survival and ameliorate cardiac dysfunction in LPS‐induced sepsis models by suppressing inflammatory cytokines, reducing apoptosis, and alleviating oxidative stress, indicating its potential as a protective agent against sepsis‐related cardiac injury [42].

Previous studies have demonstrated that several TCM‐derived compounds exert protective effects in sepsis and septic cardiomyopathy. Extracts of *Angelica sinensis* inhibit the release of the late inflammatory mediator HMGB1 by blocking its cellular translocation, thereby protecting mice from lethal endotoxemia and sepsis, even when administered 24 h after sepsis onset [43]. Sodium tanshinone IIA sulfonate has been shown to reduce cardiac injury markers and inflammatory cytokine levels, improve LV function, and increase the 18‐day survival rate in septic rats from 0% to 30%, suggesting therapeutic potential in sepsis‐induced cardiac dysfunction [44]. Similarly, salvianolic acid B alleviates septic cardiomyopathy by improving cardiac contractile function, suppressing inflammation, and attenuating mitochondrial dysfunction through activation of the mitochondrial unfolded protein response via ATF5 [45]. In addition, Tetramethylpyrazine and extracts of *Carthamus tinctorius* have been reported to induce vasodilation through endothelium‐dependent mechanisms resembling those of acetylcholine [46]. Danshen (tanshinone IIA) activates the Nrf2/HO‐1 pathway and protects against myocardial ischemia/reperfusion injury by inhibiting HDAC1, thereby reducing inflammation, apoptosis, and ferroptosis [47]. Huangqi (astragaloside IV) upregulates Nrf2 signaling and attenuates oxidative stress in sepsis models [48]. Danggui (ferulic acid) activates the Nrf2/ARE pathway and enhances antioxidant enzyme expression, thereby strengthening cellular antioxidant capacity [49]. Gegen (puerarin) alleviates LPS‐induced myocardial injury through Nrf2 activation, suppressing oxidative stress, and improving mitochondrial function and autophagy [50].

In this study, an LPS‐induced sepsis mouse model was established to evaluate the effects of YXTLD. The results showed that YXTLD treatment significantly improved cardiac function, as indicated by increased EF% and FS% compared with the LPS group (*p* < 0.01), and markedly reduced serum CK and LDH levels (*p* < 0.05), suggesting alleviated myocardial injury. Collectively, these findings indicate that YXTLD effectively restores cardiac function in sepsis‐induced cardiac dysfunction. Furthermore, in an LPS‐induced H9c2 cardiomyocyte model, as previously reported in related studies [29, 51], YXTLD enhanced cell viability, suppressed inflammation, alleviated oxidative injury, and reduced apoptosis. Mechanistically, YXTLD attenuated oxidative stress by activating the Nrf2 signaling pathway, confirming its protective effect against sepsis‐induced cardiac dysfunction. In this study, we demonstrated that YXTLD is associated with the activation of the Nrf2 pathway rather than directly activating Nrf2.

Oxidative stress is markedly elevated in the heart during sepsis, and antioxidant interventions have been shown to improve cardiac function in septic mice [52, 53]. Apoptosis is significantly increased in septic hearts, and reducing cardiomyocyte apoptosis can alleviate sepsis‐induced cardiac dysfunction [54–56]. Inflammatory cytokine release and macrophage infiltration are also evident in the myocardium of LPS‐treated mice [57]. Cardiomyocyte apoptosis, pyroptosis, and inflammation collectively contribute to the development of sepsis‐induced cardiomyopathy (SIC) [58]. Given the crucial role of Nrf2 in regulating oxidative stress and cardiac protection, the Nrf2 signaling pathway has emerged as a potential therapeutic target for sepsis‐related cardiac dysfunction. Consistent with this, our findings demonstrated that YXTLD exerts cardioprotective effects against sepsis‐induced cardiac dysfunction by reducing oxidative stress through the activation of the Nrf2 pathway.

Beyond the well‐recognized role of inflammatory cytokines, SIC involves a complex interplay of additional pathogenic mechanisms that extend beyond oxidative stress alone. First, metabolic reprogramming occurs early in septic myocardium, characterized by a shift from fatty acid oxidation to glycolysis, resulting in energy depletion and accumulation of toxic lipid intermediates [59, 60]. Second, mitochondrial dysfunction is a central driver of SIC: sepsis impairs mitochondrial electron transport chain complexes, increases mitochondrial ROS production, and promotes opening of the mitochondrial permeability transition pore (mPTP), leading to cardiomyocyte energetic failure and cell death [61, 62]. Importantly, mitochondrial ROS serve as both an upstream trigger and a downstream amplifier of Nrf2 dysregulation. Third, microcirculatory failure—including heterogeneous capillary perfusion defects, increased leukocyte–endothelial adhesion, and glycocalyx degradation—contributes to regional myocardial hypoxia and stunning independently of macrovascular function [63, 64]. Fourth, beyond apoptosis, pyroptosis mediated by the NLRP3 inflammasome and caspase‐1 has been increasingly recognized as a key mode of cardiomyocyte death in sepsis [65, 66]. While our study focused on oxidative stress and apoptosis, these additional mechanisms may also be modulated by YXTLD given that Nrf2 activation can influence mitochondrial biogenesis, metabolic flux, and inflammasome signaling.

Our findings demonstrate that YXTLD suppresses the LPS‐induced upregulation of IL‐1β, IL‐6, and TNF‐α at the transcriptional level, providing new evidence that herbal formulations can modulate inflammatory cascades in septic cardiomyopathy. By identifying Nrf2 as a key upstream regulator of both antioxidant and anti‐inflammatory responses, our study positions YXTLD as a dual‐action therapeutic agent that simultaneously targets oxidative stress and inflammation. These findings highlight a novel natural product–based strategy for modulating inflammatory pathways in the setting of critical illness. While our study focused on oxidative stress and apoptosis, these additional mechanisms may also be modulated by YXTLD given that Nrf2 activation can influence mitochondrial biogenesis, metabolic flux, and inflammasome signaling. Future studies are warranted to investigate whether YXTLD’s cardioprotective effects extend to restoring mitochondrial function, improving microcirculatory perfusion, or suppressing pyroptosis.

The limitation of our research is that (1) our study does not demonstrate a direct molecular interaction between YXTLD components and Nrf2. Whether YXTLD directly binds to Nrf2 or acts through upstream regulators (e.g., Keap1 modification) requires further investigation. (2) Loss‐of‐function experiments are still required to confirm whether the protective effects of YXTLD are specifically dependent on Nrf2 signaling. (3) Although our study demonstrated significant improvements in cardiac function and reductions in myocardial injury biomarkers following YXTLD treatment, we did not perform direct histopathological examination of the myocardial tissue, such as H&E staining to assess myofibrillar disarray, edema, inflammatory cell infiltration, or cardiomyocyte necrosis. Such analyses would provide complementary structural evidence to support our findings and should be incorporated into future studies.

## 5. Conclusion

Our findings demonstrate that YXTLD suppresses the LPS‐induced upregulation of IL‐1β, IL‐6, and TNF‐α at the transcriptional level, providing new evidence that herbal formulations can modulate inflammatory cascades in septic cardiomyopathy. By identifying Nrf2 as a key upstream regulator of both antioxidant and anti‐inflammatory responses, our study positions YXTLD as a dual‐action therapeutic agent that simultaneously targets oxidative stress and inflammation. These findings are directly relevant to the readership of Mediators of Inflammation as they highlight a novel natural product‐based strategy for modulating inflammatory pathways in the setting of critical illness.

## Author Contributions

Hainan Yang, Beijing Wu, and Die Huang designed the experiments and wrote the manuscript. Hui Ye, Weifang Yuan, and Lijiao You helped in reviewing, acquisition, analysis and interpretation of clinical data for the work. Haikuo Wang and Xueru Huang did the statistical analysis. Yaoguo Han and Ming Lei critically revised the manuscript for important intellectual content.

## Funding

This study is funded by The Scientific Research Program of Shanghai Pudong New Area Health Commission (the General Program, Grant PW2024A‐58), the National Traditional Chinese Medicine Advantageous Specialty Construction (Intensive Care Medicine), the Research on Standardized Integrated Chinese and Western Medicine Diagnosis and Treatment Protocols for Electrochemical Thermal Injury (Burns from Water and Fire) (Grant 2025BZ015), and the Clinical Emergency Medicine Center Project of Shanghai Seventh People’s Hospital (Project Number 25‐LCYZX‐01).

## Disclosure

All experiments, data processing, and manuscript writing were performed solely by the listed authors. All authors have read and approved the final manuscript.

## Ethics Statement

This study was conducted in accordance with institutional guidelines and approved by the Seventh People’s Hospital of Shanghai University of Traditional Chinese Medicine (Approval Number 2025‐AR‐009).

## Conflicts of Interest

The authors declare no conflicts of interest.

## Supporting Information

Additional supporting information can be found online in the Supporting Information section.

## Supporting information


**Supporting Information** Figure S1: Cell viability was detected by an CCK‐8 assay in indicated group.

## Data Availability

The datasets used and analyzed during the current study are available from the corresponding author upon reasonable request.
