# Supplementary figures and images for: Yangxin Tongluo Decoction Protects Against Sepsis‐Associated Cardiac Dysfunction Through Regulating Nrf2 Pathway
Source: Mediators Inflamm. 2026 Jul 23;2026:2804249. doi: 10.1155/mi/2804249 (PMC13392818; doi:10.1155/mi/2804249)

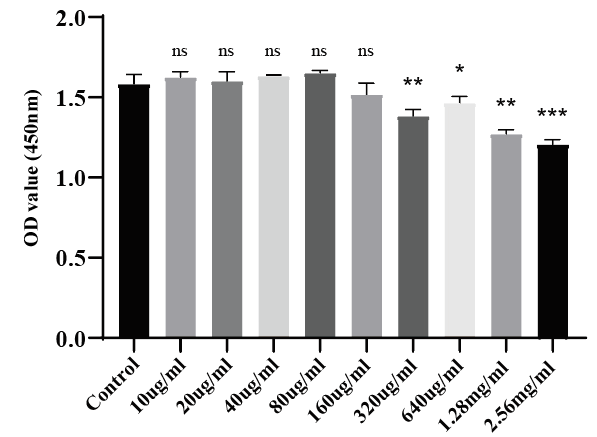


Supplementary Figure. Cell viability was detected by an CCK8 assay in indicated group.

Supplement: Supplementary file 1 — Supporting Information Figure S1: Cell viability was detected by an CCK‐8 assay in indicated group. [file MI-2026-2804249-s001.docx]
